# Intricacies in the cross talk between metabolic enzymes, RNA, and protein translation

**DOI:** 10.1093/jmcb/mjz089

**Published:** 2019-09-03

**Authors:** Yuan Lv, Muqddas Tariq, Xiangpeng Guo, Shahzina Kanwal, Miguel A Esteban

**Affiliations:** 1 Joint School of Life Sciences, Guangzhou Institutes of Biomedicine and Health and Guangzhou Medical University, Guangzhou 511436, China; 2 Key Laboratory of Regenerative Biology and Guangdong Provincial Key Laboratory of Stem Cells and Regenerative Medicine, Guangzhou Institutes of Biomedicine and Health, Chinese Academy of Sciences, Guangzhou 510530, China; 3 Laboratory of RNA, Chromatin, and Human Disease, Guangzhou Institutes of Biomedicine and Health, Chinese Academy of Sciences, Guangzhou 510530, China; 4 Guangzhou Regenerative Medicine and Health Guangdong Laboratory, Guangzhou 510005, China; 5 University of Chinese Academy of Sciences, Beijing 100049, China; 6 Institute for Stem Cells and Regeneration, Chinese Academy of Sciences, Beijing 100101, China

**Figure 1:**
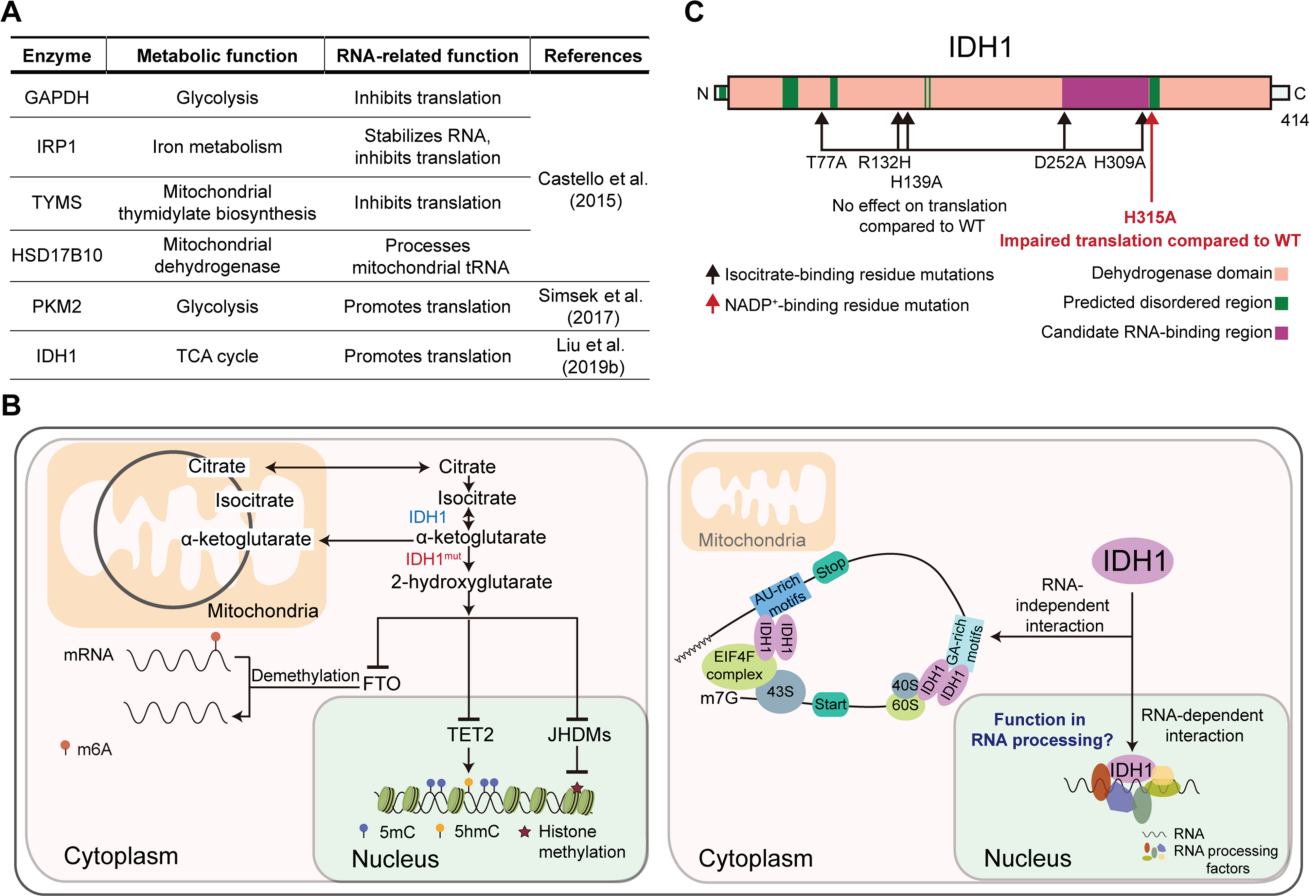
Canonical and non-canonical functions of metabolic enzymes. (**A**) Role of selected metabolic enzymes as RBPs. GAPDH: glyceraldehyde-3-phosphate dehydrogenase, IRP: iron regulatory protein 1, TYMS: thymidine synthase, HSD17B10: 17β-hydroxysteroid dehydrogenase 10; PKM2: pyruvate kinase muscle 2. (**B**) Functions of IDH1. Left panel: IDH1 and IDH1^mut^ (mutant IDH1) as metabolic regulators. IDH1 is responsible for the production of α-KG. Specific cancer-associated IDH1 mutants gain function to generate 2-HG, which in turn inhibits DNA, histone, and RNA demethylases. FTO: fat mass- and obesity-associated protein, TET2: ten–eleven translocation 2, JHDMs: JmjC domain-containing histone demethylases. Right panel: Novel function of IDH1 in protein translation. In the cytoplasm, IDH1 serves as a bridge between translation regulators and RNAs, resulting in increased cap-dependent translation. In the nucleus, IDH1 binds to multiple RNA processing factors in an RNA-dependent manner. (**C**) Effect of IDH1 substrate-binding residues on translation. Only a mutation in the NADP^+^ binding site resulted in decreased promotion of translation compared to wild-type (WT) IDH1. This residue is present in a predicted disordered region; this type of region often participates in RNA binding. Domain annotation of IDH1 is adapted from Moore et al. (2017).

The development of techniques allowing the systematic capture of the RNA-bound proteome has yielded many surprises. Among these, metabolic enzymes have been frequently detected as RNA-binding proteins (RBPs) by different profiling methodologies in various cell types (Hentze et al., 2018). Compared to previous—more simplistic—views, it is now known that cellular metabolism (not only limited to the tricarboxylic acid, TCA, cycle) is compartmentalized. In fact, metabolic enzymes translocate to the nucleus and are enriched at actively transcribed loci, where they sustain epigenetic/epitranscriptomic marks through localized metabolite production (Mews et al., 2017). It has been proposed that the interaction with nascent RNAs facilitates the anchorage of metabolic enzymes at target loci (Bao et al., 2018), and it is plausible that mature RNAs work in the same way to assist metabolic reactions in the cytoplasm. Yet, it is becoming increasingly evident that the interaction of metabolic enzymes with RNA also has metabolism-independent functions, and protein translation is emerging as a key missing nexus (Figure 1A). For example, the glycolytic enzyme pyruvate kinase muscle 2 (PKM2) promotes protein translation through simultaneous binding to target mRNAs and ribosomal components (Simsek et al., 2017). Thus, deeper understanding of the relationship between RNA and metabolic enzymes is necessary for gaining a complete model of metabolism and other cellular functions as a whole.

Isocitrate dehydrogenases (IDHs) compose a family of enzymes (IDH1–IDH3) catalyzing the reversible conversion of the TCA product isocitrate into α-ketoglutarate (α-KG) (Figure 1B, left), a reaction that involves the transformation of NADP^+^ into NADPH. Of note, α-KG is not only the substrate for subsequent TCA reactions, ultimately aiming to produce ATP, but also a cofactor for dioxygenases including DNA, histone, and RNA demethylases (Cloos et al., 2008; Fan et al., 2015). Moreover, NADPH is the universal electron donor in reductive biosynthesis and, so, an essential regulator of cellular redox balance. Interestingly, the expression of IDH enzymes (in particular IDH1 and IDH2) is frequently altered in multiple types of cancer, and a number of mutations have been reported too (Tommasini-Ghelfi et al., 2019). Some IDH1/2 mutations result in the production of the potential oncometabolite 2-hydroxyglutarate (2-HG), which at higher concentrations inhibits dioxygenases (Dang et al., 2010; Lemonnier et al., 2016), supporting the idea that IDH alterations promote cancer by changing the epigenetic/epitranscriptomic balance. Yet, preclinical studies with inhibitors of mutant IDH1 have shown variable effects on cancer progression, and it has been reported that 2-HG can inhibit glioma cell proliferation rather than enhance it (Su et al., 2018). These observations imply that alterations of other, metabolic-independent, functions of IDH enzymes might be more relevant contributors to disease.

Previously, Liu et al. (2019a) used a protein microarray approach to identify IDH1 as an RBP. Accordingly, cross-linking immunoprecipitation associated to high-throughput sequencing (CLIP-sequencing) in mouse embryonic stem cells (mESCs) demonstrated that IDH1 binds thousands of RNAs including mRNAs encoding proteins involved in transcriptional regulation, RNA metabolism, and the cell cycle. IDH1 had also been identified as a putative RBP in an oligo(dT) capture study using HeLa cells (Castello et al., 2012). In this issue of *Journal of Molecular Cell Biology*, Liu et al. (2019b) show that IDH1 promotes translation of IDH1 target mRNAs in mESCs in a cap-dependent manner, a mechanism that involves binding of the EIF4F complex to the 7-methylguanosine cap structure at the 5′ end of mRNA (Figure 1B, right). Importantly, IDH1 target mRNAs comprise many unstable mRNAs and/or encode unstable proteins, suggesting that the interaction with IDH1 fine-tunes translation to adjust protein levels. The authors characterized the IDH1-interacting proteome of mESCs in different compartments (nucleoplasm, chromatin, and cytoplasm), detecting enrichment in translational regulators (including members of the EIF4F complex) in the cytoplasmic compartment and RNA processing factors in the other two. Purification of IDH1 using polysome fractionation further confirmed that IDH1 interacts with the translational machinery in the cytoplasm. Surprisingly, treatment with RNase A demonstrated that IDH1–protein interactions in the nucleoplasm are RNA dependent, whereas those on chromatin and in the cytoplasm are mainly RNA independent. This suggests that the interaction of IDH1 with RNA processing factors in the nucleus is important for determining the cytoplasmic effects on protein translation, perhaps by controlling RNA structure. The authors also introduced different mutations in IDH1 substrate-binding residues including the naturally occurring cancer mutation R132H. However, they observed that mutations altering isocitrate binding have no detrimental effect on protein translation, whereas a mutation (H315A) affecting NADP^+^ binding significantly impaired the ability to promote protein translation compared to wild-type IDH1 (Figure 1C). The latter is a further demonstration of the separation between the metabolic and protein translation functions of IDH1.

In summary, Liu et al. (2019b) have demonstrated a novel role for IDH1 in fine-tuning protein translation independently of its catalytic activity, strengthening the growing body of evidence that metabolic enzymes, RNA, and protein translation are all intrinsically connected. In the future, it will be important to study whether and how IDH1 regulates other key steps of translation besides initiation and to explore this non-canonical function in cancer and cellular responses. From a wider perspective, it is interesting to speculate that metabolic enzymes are also involved in other cell metabolism-independent aspects of RNA regulation in addition to translation.

*[Work in this topic in the Esteban's laboratory is supported by the National Key Research and Development Program of China (2018YFA0106903).]*
